# An improved independent component analysis model for 3D chromatogram separation and its solution by multi-areas genetic algorithm

**DOI:** 10.1186/1471-2105-15-S12-S8

**Published:** 2014-11-06

**Authors:** Lizhi Cui, Josiah Poon, Simon K Poon, Hao Chen, Junbin Gao, Paul Kwan, Kei Fan, Zhihao Ling

**Affiliations:** 1 School of information technologies, University of Sydney, Sydney, NSW 2006, Australia; 2 School of Computing and Mathematics, Charles Sturt University, Bathurst, NSW 2795, Australia; 3 School of Science and Technology, Univeristy of New England, Armidale, NSW 2350, Australia; 4 Key Laboratory of Advanced Control and Optimization for Chemical Processes, Ministry of Education, East China University of Science and Technology, Shanghai, 200237, China

## Abstract

**Background:**

The 3D chromatogram generated by High Performance Liquid Chromatography-Diode Array Detector (HPLC-DAD) has been researched widely in the field of herbal medicine, grape wine, agriculture, petroleum and so on. Currently, most of the methods used for separating a 3D chromatogram need to know the compounds' number in advance, which could be impossible especially when the compounds are complex or white noise exist. New method which extracts compounds from 3D chromatogram directly is needed.

**Methods:**

In this paper, a new separation model named parallel Independent Component Analysis constrained by Reference Curve (pICARC) was proposed to transform the separation problem to a multi-parameter optimization issue. It was not necessary to know the number of compounds in the optimization. In order to find all the solutions, an algorithm named multi-areas Genetic Algorithm (mGA) was proposed, where multiple areas of candidate solutions were constructed according to the fitness and distances among the chromosomes.

**Results:**

Simulations and experiments on a real life HPLC-DAD data set were used to demonstrate our method and its effectiveness. Through simulations, it can be seen that our method can separate 3D chromatogram to chromatogram peaks and spectra successfully even when they severely overlapped. It is also shown by the experiments that our method is effective to solve real HPLC-DAD data set.

**Conclusions:**

Our method can separate 3D chromatogram successfully without knowing the compounds' number in advance, which is fast and effective.

## Background

**Figure 1:**
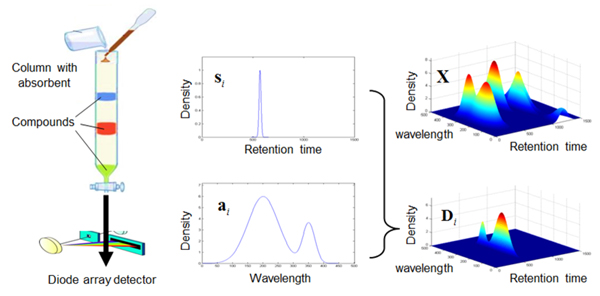
**The principle of HPLC-DAD dataset**. The vector of $s_{i}$ represents chromatogram peaks and the vector of $a_{i}$ represents spectra. The matrix of $D_{i}$ represents 3D dataset containing only one compound, which is formed by $a_{i}\times s_{i}^{T}$. The matrix of X represents the dataset containing more than one compound.

For thousands of years, plants have played a dominant role in the development of sophisticated traditional herbal medicine (HM) systems [1,2]. And nowadays, HM has also attracted much interest of both patients and scientists [3]. However, herbal medicines are extracted with boiling water during the decoction process, which makes it very difficult to realize quality control [4]. In 1991 [5], the World Health Organization (WHO) accepted chromatography fingerprint, which reflects the complex chemical composition of the analyzed sample based on spectroscopic, chromatographic or electrophoretic techniques [6], as a methodology for the assessment of natural products. But, two disadvantages exist for chromatography fingerprint: it relies on retention time which is not stable; it is chosen from only one specific wavelength which misses much information from other wavelength. So, 3D chromatogram generated by High Performance Liquid Chromatography-Diode Array Detector (HPLC-DAD) was researched widely [7-10]. The construction of HPLC-DAD dataset is illustrated in Figure 1, in which there are three compounds contained in the solution for example.

A drop of sample is injected at the top of a column with absorbent. A cup of solvent, same as that in the sample, carries the sample through the column. Different compounds will receive different resistance when they go through the column. Given an ultraviolet detector at the bottom of the column, a chromatogram peak represented by $s_{i}\left(i=1,2,3\right)$ is formed to reflect the concentration for corresponding compound. If using a DAD for detection, which has more than one thousand channels to detect multi-wavelength simultaneously, besides chromatogram peaks of the outflowing compounds, spectra represented by $a_{i}\left(i=1,2,3\right)$ will also be recorded. The matrices of $D_{i}$ and **X **represent *i^th ^*compound and the mixture of all the compounds respectively. Their relationship is given as

(1) $$X=\overset{n}{\underset{i=1}{\sum }}D_{i}=\overset{n}{\underset{i=1}{\sum }}a_{i}\times s_{i}^{T}=\left[a_{1},a_{2},\cdots \;,a_{n}\right]\times \left[\begin{array}{c} s_{1}^{T}\\ s_{2}^{T}\\ \vdots \\ s_{n}^{T} \end{array}\right]=AS$$

where, the variable of *n *is the number of compounds contained in the solution, which equals to 3 for the example in Figure 1.

There are many methods to separate **X ** in (1), such as evolving factor analysis (EFA)[11], heuristic evolving latent projections (HELP)[12], window factor analysis (WFA)[13], orthogonal projection resolution (OPR)[14], evolving window orthogonal projections (EWOP)[15], iterative target transformation factor analysis (ITTFA)[16], alternating regression (AR)[17], parallel factor analysis (PARAFAC1/2)[18], multivariate curve resolution-alternating least squares (MCR-ALS)[19] and interactive self-modelling mixture analysis (SIMPLISMA)[20], alternating trilinear decomposition (ATLD)[21] and immune algorithm (IA)[22]. However, all these method need the number of the compounds to be known in advance. And the method to obtain the compounds' number is based on Eigenvalue, which will miss small peaks especially when noise is severe. Recently, Independent Component Analysis (ICA)[23] was introduced in this field, which considered compounds and noises as independent components. But two disadvantages existed: 1) noises was considered as independent components, which gave unexpected and useless information in the results; 2) identifying compounds from noises after separating all the independent components was still needed.

In order to extract compounds directly from the data set, this paper proposed a parallel model of Independent Component Analysis constrained by Reference Curves (pICARC) and its solution by multi-areas Genetic Algorithm (mGA). In section 2, the principle of pICARC and mGA were proposed. In section 3, simulations and experiments were provided to show the performance of our method. Finally, conclusions and future works were summarized in section 4.

## Methods

**Figure 2:**
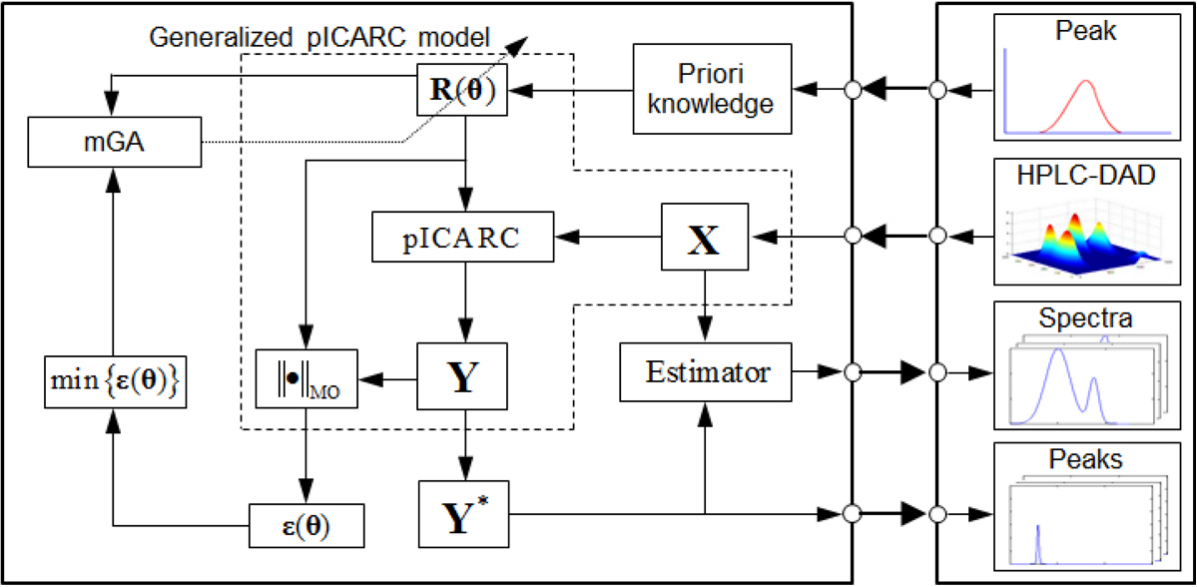
**The structure of our method**. R(θ) is Reference Curve (RC), **Y **is Calculated Curve (CC), ${∥•∥}_{\text{MO}}$ is Measurement Operator (MO) to give the distance ε(θ) between R(θ) and **Y **

The principle of our method is illustrated in Figure 2. The left thick box is the mathematical part, and the right thick box are corresponding data sets in the reality. Firstly, we construct a kind of Reference Curve (RC) R(θ) with different parameter of *θ * based on the priori knowledge. Then inputting R(θ) and **X **into the pICARC model, Calculated Curves (CCs) **Y **will be obtained. The distances, ε(θ), between R(θ) and **Y **are calculated by the Measurement Operator (MO) ${∥•∥}_{\text{MO}}$. Combining all the elements contained in the dash polygon together, it is called generalized pICARC model, which has converted the separation problem to a multi-parameter optimization issue. Next, what is needed is to find the parameters of $\theta^{*}$, which minimizes the value of ε(θ).

In this paper, the algorithm of mGA, which is proposed with reference to the features of R(θ), is used to search $\theta^{*}$. After all the $\theta^{*}$ have been found, $Y^{*}$ will be obtained, whose row vectors are the chromatogram peaks for individual compounds. By using an estimator, the spectra of the compounds will be obtained. The priori knowledge has been introduced within our method. What the user needed to do is just to input your data set**X **.

Following, the priori knowledge about chromatogram peaks, the pICARC model, MO, mGA and the estimator will be introduced one by one.

### The priori knowledge

**Figure 3:**
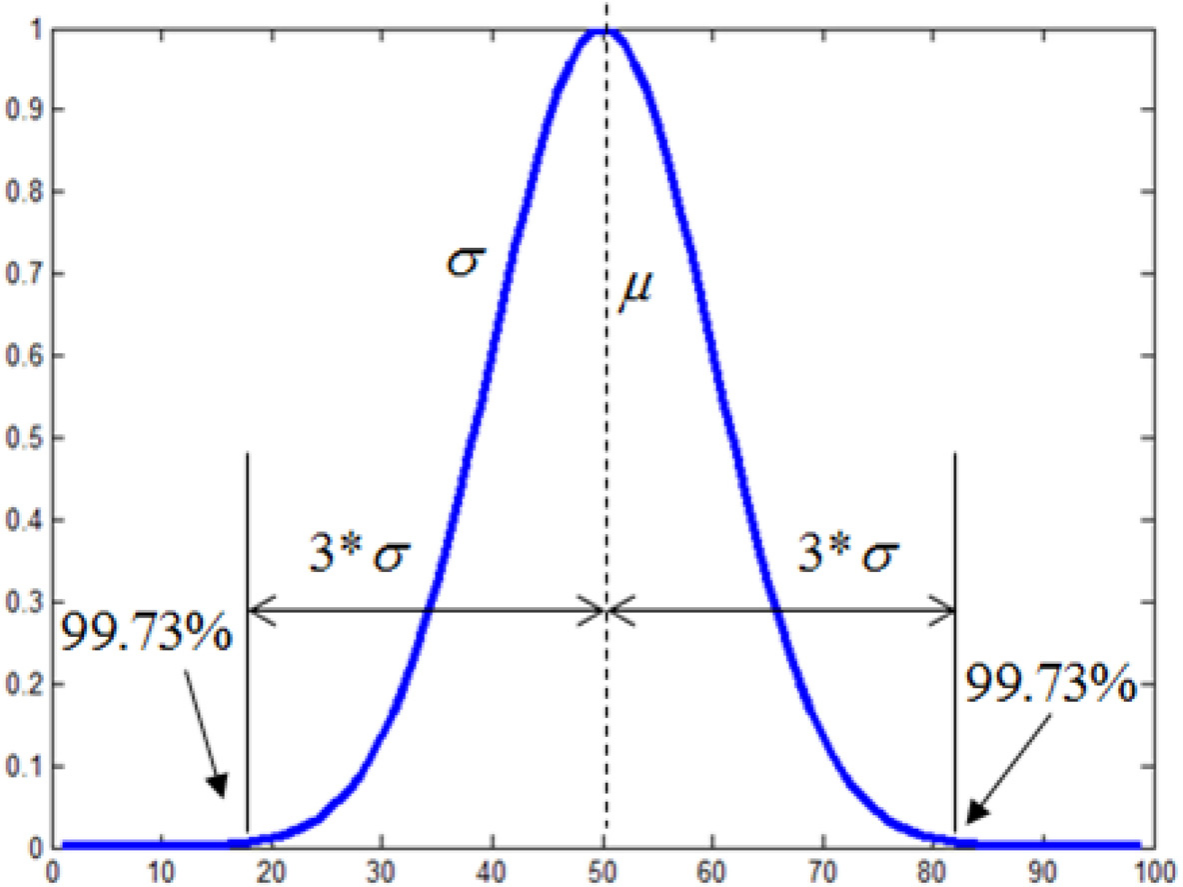
**Illustration of Gaussian curve**.

According to the physical principle of chromatography, each peak of chromatogram looks like certain curves such as Gaussian curve, Log-normal curve, Gamma curve and Weibull curve, etc. [24], among which, the Gaussian function was used prevalently for simulating chromatogram peaks in many relevant researches. [25,26] So, we will use Gaussian curve, which is illustrated in equation (2) and Figure 3, in our research. There are two parameters: *μ * and *σ *.

(2) $$y=\text{exp}\left[-\frac{{\left(x-\mu \right)}^{2}}{2*\sigma^{2}}\right]$$

where, the factor of $1/\sqrt{2\pi }\sigma$ is removed to set the maximum value to 1. The amplitude information will be found in the spectra.

The range of the parameter *μ * is decided by the data set, whose minimum value is $\mu_{1}=1$ and maximum value is $\mu_{2}=column\left(X\right)$. According to the quantile of 99.73%, we have the following inequality

(3) $$\sigma <\frac{\mu_{2}-\mu_{1}+1}{6}$$

### pICARC model

The model of ICA is represented as (4)

(4) $$X=\left[x_{1},x_{2},\cdots \;,x_{t}\right]=A\times S=A\times \left[s_{1},s_{2},\cdots \;,s_{t}\right]=A\times \left[\begin{array}{c} s_{1}^{T}\\ s_{2}^{T}\\ \;\vdots \\ s_{n}^{T} \end{array}\right]$$

where **X ** are the observation vectors; **A **is the mixed matrix; **S ** are the source vectors; the subscript of ***t ***is the number of the samples in **A **and **S **. The purpose of this model is to obtain **A **and **S ** only based on **X ** under four assumptions[27]. According to the separability theorem of ICA[28], we could trust ICA to separate HPLC-DAD data set as long as the number of the wavelength is greater than the number of the compounds. In 1999, the algorithm of fastICA was proposed to solve (4)[29,30], of which the parallel form is shown as

(5) $$\left\{\begin{array}{c} \text{max}\;E\left\{G\text{(}B^{\text{T}}\tilde{\text{X}}\text{)}\right\}=E\left\{G\text{(}\left[\begin{array}{c} \overset{T}{\underset{1}{b}}\\ \overset{T}{\underset{2}{b}}\\ \vdots \\ \overset{T}{\underset{n}{b}} \end{array}\right]\left[{\tilde{\text{x}}}_{1},{\tilde{\text{x}}}_{2},\cdots \;,{\tilde{P\text{x}}}_{n}\right]\text{)}\right\}\\ \text{subject to }E\left\{{\text{(}b_{i}^{T}\tilde{\text{x}}\text{)}}^{\text{2}}\right\}={∥{\text{b}}_{i}∥}^{2}=1,\;\left(i=1,2,\cdots \;,n\right) \end{array}\right)$$

where, $b_{i}$ is a m-dimensional vector such that$E\left\{{\left(b^{T}\tilde{\text{x}}\right)}^{2}\right\}=1$, $\tilde{\text{X}}$ is a matrix preprocessed from**X **, $\text{G}\left(\text{x}\right)=1/4∙\left({\text{x}}^{4}\right)$ is a nonlinear function. Applying KKT condition [31] and Newton method [32], the iterative equation of **B ** is shown as

(6) $$\left\{\begin{array}{c} B^{+}=E\left\{\tilde{\text{X}}g\left(B^{T}\tilde{\text{X}}\right)\right\}-E\left\{g^{\prime }\left(B^{T}\tilde{\text{X}}\right)\right\}B\\ B^{+}={\left(BB^{T}\right)}^{-1/2}B \end{array}\right)$$

where, g(∙) is the first derivative of G(∙).

As mentioned in the introduction, two major problems were found when applying the ICA model to HPLC-DAD data set. Therefore, suitable modification about ICA based on the priori knowledge of chromatogram peaks is needed to constrain the shape of the source signals. What should be noted is that the variable used for calculation in ICA model is the $\tilde{\text{X}}$, which generates a signal of ${y^{\prime }}_{i}$ shown in equation (7). However, the curve that should be constrained is $y_{i}$, which is a calculated signal to approximate $s_{i}^{T}$. The $D_{w}$ is the whitening matrix. There is a difference between ${y^{\prime }}_{i}$ and $y_{i}$, which is caused by pre-processing.

(7) $$\left\{\begin{array}{c} {y^{\prime }}_{i}=\left[{y^{\prime }}_{i1},{y^{\prime }}_{i2},\cdots \;,{y^{\prime }}_{it}\right]=b_{i}^{T}\tilde{X}=b_{i}^{T}\left[{\tilde{x}}_{1},{\tilde{x}}_{2},\cdots \;,{\tilde{x}}_{t}\right]\\ y_{i}=\left[y_{i1},y_{i2},\cdots \;,y_{it}\right]=b_{i}^{T}D_{w}X=b_{i}^{T}D_{w}\left[x_{1},x_{2},\cdots \;,x_{t}\right] \end{array}\right)$$

In order to avoid introducing a new variable, which should represent the difference between ${y^{\prime }}_{i}$ and $y_{i}$, the model is constructed as

(8) $$\left\{\begin{array}{c} \text{max}\left[E\left\{G\left(\overset{T}{\underset{}{\bar{B}}}\tilde{X^{\prime }}\right)\right\}-{∥{\bar{B}}_{}^{T}\tilde{X^{\prime \prime }}-R\left(\theta \right)∥}_{\text{MO}}\right]\\ R\left(\theta \right)={\left[r_{1}\left(\theta \right),r_{2}\left(\theta \right),\cdots \;,r_{n}\left(\theta \right)\right]}^{T}\\ {\bar{B}}^{T}=\left[B^{T},d\right]=\left[{\left[b_{1},b_{2},\cdots \;,b_{n}\right]}^{T},d_{1\times n}\right]\\ \tilde{X^{\prime }}={\left[{\tilde{X}}_{}^{T},0\right]}^{T}\\ \tilde{X^{\prime \prime }}={\left[{\tilde{X}}_{}^{T},1\right]}^{T}\\ E\left\{{\left(b_{i}^{T}\tilde{x^{\prime }}\right)}^{2}\right\}=E\left\{{\left({\bar{b}}_{i}^{T}\tilde{x^{\prime }}\right)}^{2}\right\}={∥b∥}^{2}=1 \end{array}\right)$$

where **d ** represents the differences existing in the equation (7). The equation (8) is called pICARC.

### Measurement operator

The purpose of MO is to measure the distance between $y_{i}$ and $r_{i}\left(\theta \right)$. Here, the vector of $\varepsilon \left(\theta \right)=\left[\varepsilon_{1}\left(\theta \right),\varepsilon_{2}\left(\theta \right),\cdots \;,\varepsilon_{n}\left(\theta \right)\right]$ contains all these distances. We gave the definition of $\varepsilon_{i}\left(\theta \right)$as

(9) $$\varepsilon_{i}\left(\theta \right)={∥y_{i}-r_{i}\left(\theta \right)∥}_{2}^{2}=\underset{j}{\sum }{\left[y_{i}\left(j\right)-r_{i}\left(j;\theta \right)\right]}^{2}$$

where, *j *represents every element in the vectors of $y_{i}$ and $r_{i}\left(\theta \right)$

### Multi-area genetic algorithm

GAs are a family of computational models inspired by evolution. These algorithms encode a potential solution to a specific problem on a simple chromosome-like data structure, and apply recombination operators to these structures in such a way as to preserve critical information. Usually, GAs are used to find one global optimal point in the searching plane. But in our problem, several points in the μ-σ plane should be found as solutions simultaneously. In order to search multi optimal points, we propose an algorithm named multi-areas Genetic Algorithm (mGA).

Areas are circles which are composed of chromosomes that are closed to one another, where the candidate solution will be found. If the fitness of one solution is much better than the others, the area around it will have better fitness as well. This will lead many elites assemble into this area. In order to balance the number of elites among all the areas, emigrant and immigrant policy are adopted. Except limited chromosomes left in every area, the others will be selected as emigrants, which will keep balance among population in all areas. Immigrant policy under certain criterion introduces new chromosomes into all areas.

**Figure 4:**
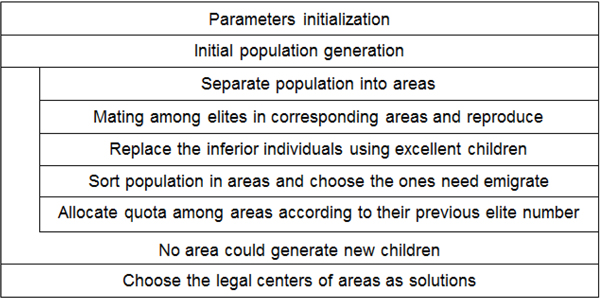
**Flow chart of mGA**.

The flow chart of mGA is illustrated in Figure 4. Firstly, parameters, which will be depicted in step 1), are initialized. Secondly, initial population is generated. Then, multi-areas have been formed among the population. Each of these areas has a center and a radius. Only in the areas, whose radius are big enough, the elite chromosomes have the right to match with each other and generate children. Children with high fitness values will replace the inferior individuals. Only a certain number of those top ranking chromosomes in an area will be kept for the next generation, the others will be selected as emigrants to avoid too many chromosomes converging in one dominant area. Later, the emigrants will be allocated into different areas under certain law, which will be descripted in step 6).

After the migration process, new generation has been formed. New multi-areas will be separated among the new generation again. The iteration continues until there is no area with the size big enough to generate children. Finally, only several areas left, which contain the candidates for solution. Solutions are found according to the decision as described in step 7).

1) Parameters initialization: The major parameters include the number of the population, the number of elites, length of the chromosome and the fitness function. The first three are decided according to the size of the search plane. The fitness of every chromosome is given by equation (9). Here, smaller the fitness is, more superior the chromosome is.

2) Population initialization: The population is equal to the row number of R(θ). In order to get initial population with better fitness, two steps were adopted. Firstly, the search space is separated into several sub spaces equally, and maximum number of chromosomes is randomly generated in every sub spaces. Then, top ones according to their fitness value are selected as initial population.

3) Multi-areas separation: Multi-areas are formed among the population according to following steps: (a) select the chromosome with best fitness as the center of an area; (b) give an user-defined radius and draw a circle; (c) calculate the distance between center and the other chromosomes contained in this area, update the radius of this area with the largest distance; (d) for chromosomes not belonging to a specific area, repeat Step (a) to (d) until no more chromosome left.

Some of these circles (areas) may overlap with each other. For those circles with severe overlapping, they should be merged as a big area for immigration; this will be further described in step 6).

4) Mating and reproduction: Every elite finds a mate which has the highest hamming distance, i.e. the number of different bits between them, by the sequence ordered of fitness. New chromosomes, the children, are generated by crossing every different bit between the mated pairs. If a child has better fitness value than any one of its parent, it will be classified as excellent child, which will be reserved; otherwise, it will be dropped.

5) Select migrants: Only limited chromosomes, whose number is user-defined depending on application, according to their fitness will stay in one area, the others are selected as emigrants. In this step, only the number of the migrants is decided. The generation of immigrants will be descripted in step 6).

6) Migrant quota among areas allocation: For every circle (area), more elites in the previous generation, less immigrants will be allowed, to avoid too many elites in one area. If two areas are severely overlapped, they will be merged as one big area to receive immigrants. Otherwise, the quota will be unfair because of the overlap. Take the experiments in this paper as an example. There is a severe overlap between area 1 and area 3. And there are 23 elites in area 1 and 1 elite in area 3. If the area 1 and area 3 are not combined as one, there will be a small number of migrants for area 1 but many for area 3. The immigrant in area 3 can also be considered as immigrant for area 1 because of the overlap. There is also an overlap between area 1 and area 5, but the center of area 5 is at the boundary of the μ-σ plane, which is ignored for further evolution.

The chromosomes with the same number as the initial population will be generated randomly for every circle (area), but only top ones decided by the quota are selected as immigrants.

7) Find solution: As the algorithm proceeds, the radii of the areas will become smaller. When all the radii become small enough, the program ends. The center of each area is the candidate solution. If the center is a local minimum, it will be selected as a solution.

### Estimator

After obtaining all the chromatogram peaks, $Y^{*}\approx S$, by solving equation (8), the spectra can be estimated with $Y^{*}$ and **X **with considering the noise contained in **X **. For simplicity, we ignore the noise in this paper to calculate spectra by

(10) A=X*pinv(S)

Where the pinv(∙) is the pseudo inverse function. Equation (10) is derived from equation (1) directly.

## Results and discussion

In this section, a group of simulations were given to explain the principle of our method. Then several experiments on a real HPLC-DAD data set were given to demonstrate the practicability of our method. Two criteria were used to evaluate our method: 1) to see whether the compounds' number found by our method was right; 2) to see the errors between the true spectra and calculated spectra.

### Simulations and discussion

**Figure 5:**
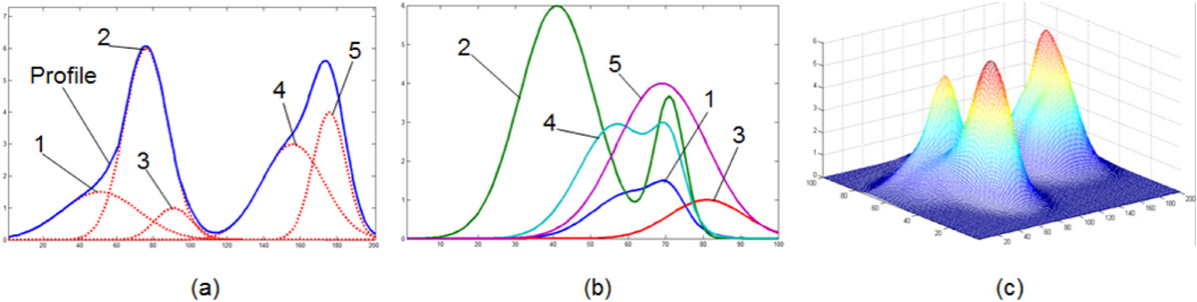
**Dataset for simulation**. (a) Chromatogram peaks for compounds. (b) Spectra for compounds. (c) 3D chromatogram.

**Figure 6:**
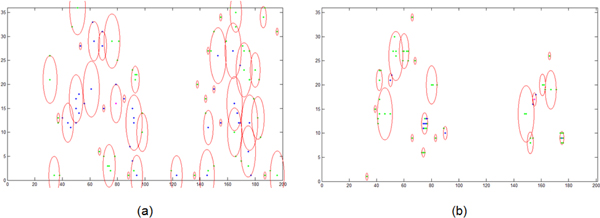
**Distribution of the parameters**. The abscissa is *μ *, the ordinates is *σ *.(a) Distribution of the initial parameters. (b) Distribution of the final parameters.

As illustrated in Figure 5, five compounds' chromatogram peaks, which are represented by different parameters of (μ,σ) respectively, are constructed in the simulation dataset. The parameters for the compounds' chromatogram peaks from 1^st ^to 5^th ^are: (50, 21), (75, 12), (90, 10), (155, 17) and (175, 9) respectively. These initial parameters' distribution on the μ-σ plane is shown in Figure 6. There are many areas for the initial parameters, but few areas for the final parameters. The program is available from the corresponding author.

From the simulation, we can see:

(1) The method proposed in this paper could separate 3D chromatogram into chromatogram peaks and spectra effectively without know the compounds' number in advance even severe overlap exist. The pICARC model transformed the separation problem to a multi-parameter optimization issue, which could be solved by swarm intelligent algorithm. The algorithm of mGA could find all the solutions simultaneously.

(2) Sometimes, the result given by this method was incorrect. This is because that the chromosome is initialized randomly, which will cause undetermined situation. This problem can be solved by running program multiple times and compare the candidate results to obtain the final results. Ten times of simulation have been done in this simulation and seven times have the correct result, which is list in Table 1.

**Table 1:** Correct results for the simulation.

Areas	Center	Radius	NumE	NumP	ErrorC	ErrorS
1	(155,17)	1	6	6	6.8102e-9	1.8503e-7
2	(75,12)	1.4142	2	5	1.0044e-8	1.1303e-8
3	(90,10)	1	21	26	1.0864e-8	1.372e-7
4	(175,19)	3.1623	4	6	1.6714e-8	5.1367e-8
5	(50,21)	1	1	3	2.4052e-8	1.4096e-7

Column of NumE is the elites' number contained in this area. Column of NumP is the population's number contained in this area. Column of ErrorC is the ε(θ) between the reference curves with the center as parameters and the chromatogram peaks. Column of ErrorS is the error between the calculated spectra and the true spectra.

(3) The implementation of the mGA is fast. Among ten times of simulation, the slowest one took 11 steps and 4.3652 seconds.

### Experiments and discussion

**Figure 7:**
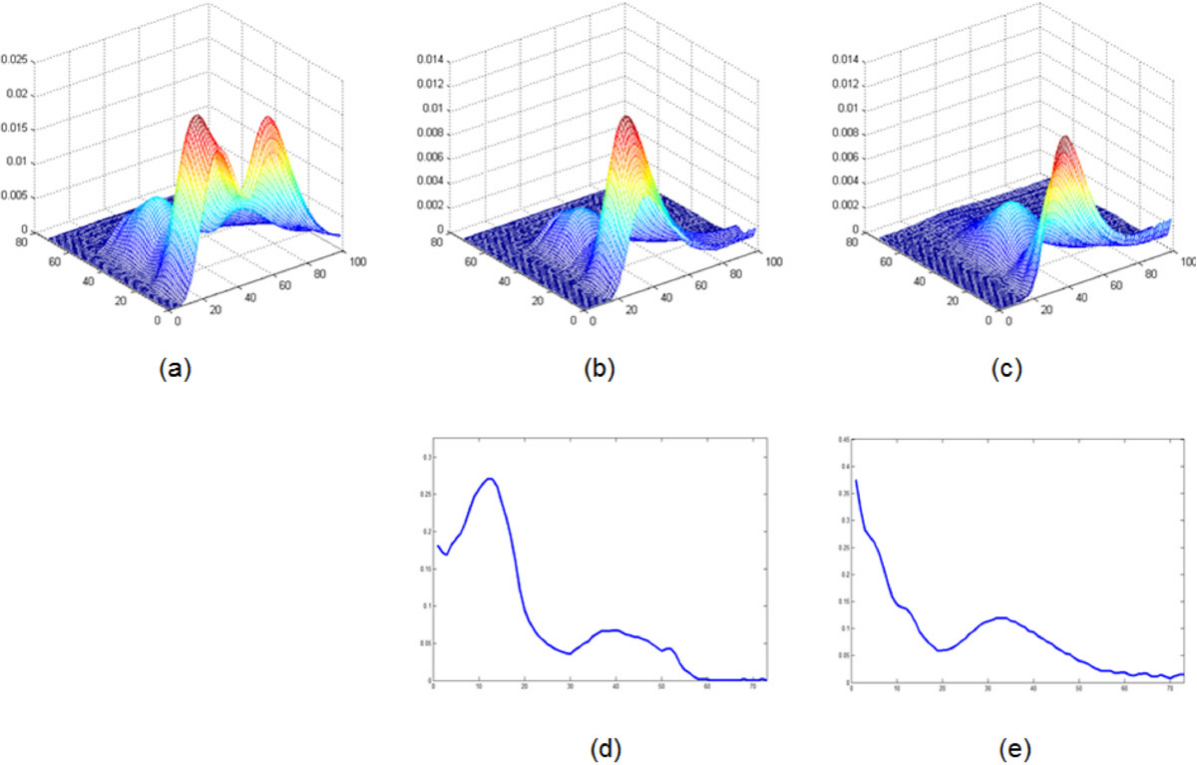
**HPLC-DAD data set used in the experiments**. (a) 3D chromatogram contains all the three compounds. (b) 3D chromatogram contains only the first compound. (c). 3D chromatogram contains only the second compound. (d) The spectrum of the first compound. (e) The spectrum of the second compound.

The program of experiment is available for free from the corresponding author. The data set of "adataset.mat", which is used in the experiment, can be downloaded from http://www.mcrals.info for free [33]. The data set is illustrated in Figure 7. The data set is a three-compound system with two pesticides identified and one unknown interferent. The three-way data set is formed by one matrix with the three compounds and two matrices of standards with one known compound.

**Figure 8:**
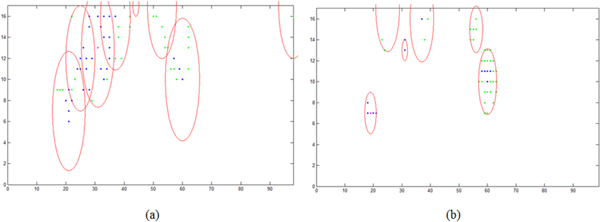
**Distribution of population**. (a) The initial population. (b) The final population.

**Figure 9:**
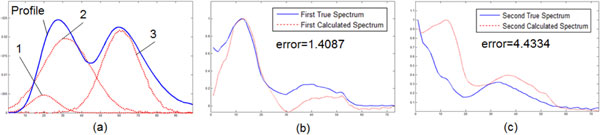
**Results of the experiment**. (a) Three calculated chromatograms. (b) Compare between first true spectrum and first calculated spectrum. (c) Compare between second true spectrum and second calculated spectrum.

Ten experiments were run totally. As the population initialization was randomly, we just list the initial population and initial multi-areas for the first experiment in Table 2. Among the ten experiments, eight gave the same results: (19, 7), (31, 13) and (60, 10). The initial population distribution and the final population distribution of one experiment are illustrated in Figure 8. The calculated results are illustrated in Figure 9.

**Table 2:** Initial population and initial multi-area for the first experiment.

Areas	Center	Radius	Error	NumE	NumP	Big area
1	(31,13)	5.6569	0.0045	23	25	1
2	(21,7)	5.6569	0.0062	8	13	2
3	(25,12)	5	0.0099	1	2	1
4	(60,10)	5.8310	0.0100	3	11	3
5	(37,16)	5.0990	0.0108	1	8	0
6	(53,17)	5	0.0173	0	9	0
7	(44,17)	1	0.0180	0	2	0
8	(98,17)	5	0.0205	0	3	0

Column of error is the ε(θ) between the reference curves with the center as parameters and the chromatogram peaks. Column of NumE is the elites' number contained in this area. Column of NumP is the population's number contained in this area.

From the results, we can see:

(1) The method proposed in this paper can separate true HPLC-DAD data set into chromatogram peaks and spectra without know the compounds' number in advance. In the ten experiments, eight gave correct results. And the maximum step and time cost are 16 and 2.6854 seconds.

(2) In (a) of Figure 9, the Profile is the projection of the data set along the axis of wavelength. And the chromatogram peaks are products of the calculated curves by the maximum value of the corresponding calculated spectra. The reason that the second peak is higher than the profile is that there is some values in the spectra are negative. The reason for the negative will be discussed in next item.

(3) In (b) and (c) of Figure 9, there are still errors between the calculated spectra and true spectra. This could be caused by following two reasons: noise existed in the data set; difference between the reference curve and true chromatogram peaks. For the first reason, the estimator, shown in equation (10) should be improved. For the second reason, more detailed reference curve should be proposed to replace equation (2).

## Conclusions

In this paper, the pICARC model and its solution by mGA were proposed. A priori knowledge of chromatogram peaks is introduced into ICA model. And the shape of the chromatogram peaks, which are the source signals in the ICA model, is constrained with certain kind of function with parameters. Because the Gaussian curve is used widely in relevant field, we use Gaussian curve to constrain the shape of the chromatogram peaks. In order to solve this model, which contained several objective points in the μ-σ plane, we modified the algorithm of GA to propose the algorithm of mGA. This algorithm separated all the population into multi-areas according to their fitness and distances from each other. Immigrant policy was adopted to keep the variety of the population. In order to avoid gathering too many elites into one dominant area, the immigrants were allocated according to the former elite number of the destination area. Finally, simulations and experiments were done to prove the performance of our model and its solution algorithm. In this section, conclusions were summarized firstly, and then future works were prospected.

### Conclusions

(1) The pICARC model transformed the separation problem of HPLC-DAD data set to a multi parameters optimization issue, which can be solved by abundant optimization algorithms.

(2) By introducing a priori knowledge of chromatogram into the ICA model, only the useful signals will be picked out from the mixed data set. It is not necessary for us to know the compounds' number in advance or to discriminate the signal from noises after finding out all the independent components. This means that this model improves the accuracy as well as saves the time for calculation.

(3)The algorithm of mGA is a useful method to search multiple objective points in the μ-σ plane simultaneously. The information of chromosome' fitness and their distance from each other were used to cluster them into multi-areas. Immigrant policy and quota allocation law were made to keep the variety for every areas. Genetic operators were used to keep the evolution among areas.

### Future works

According to the discussions in section 3, three major works are needed to be done in the future:

(1) Improved estimator should be developed to eliminate the effect caused by noise existing in the data set. The estimator used in this paper, shown by equation (10), ignores the noise. So there are errors existing in the results.

(2) More accurate reference curves should be introduced into the pICARC model. The other functions referred in section II should be used to see whether better results could be obtained. In reality, the chromatogram curve maybe more complex than the functions referred in this paper, new reference curves with more parameters could be proposed based on the experiments for out model.

(3) Facing new reference curves, which could have more parameters than that used in this paper, new optimization algorithm should be developed to fit the new parameters space.

## List of abbreviations

**HPLC-DAD: **High Performance Liquid Chromatography-Diode Array Detector

**ICA: **Independent Component Analysis

**pICARC: **parallel Independent Component Analysis constrained by Reference Curve

**GA: **Genetic Algorithm.

**mGA: **multi-area Genetic Algorithm.

## Competing interests

The authors declare that they have no competing interests.

## Authors' contributions

LC designed the pICARC model, designed the mGA algorithm and drafted the manuscript. JP and SKP supervised the manuscript, conceived of the whole method and revised the manuscript carefully. HC participated in the revision of this manuscript. JG and PK participated in the design of pICARC model and helped to draft the manuscript. KF supervised the manuscript from pharmacy view. ZL participated in the design of the simulations and experiments. All authors read and approved the final manuscript.

## Declarations

This supplement has not been supported by sponsorship.

This article has been published as part of *BMC Bioinformatics *Volume 15 Supplement 12, 2014: Selected articles from the IEEE International Conference on Bioinformatics and Biomedicine (BIBM 2013): Bioinformatics. The full contents of the supplement are available online at http://www.biomedcentral.com/bmcbioinformatics/supplements/15/S12.
